# A Co-Located sEMG-pFMG Dataset for Hand Gesture Recognition Under Varying Arm-Position Conditions

**DOI:** 10.3390/s26144626

**Published:** 2026-07-21

**Authors:** Shen Zhang, Hao Zhou, Rayane Tchantchane, Gursel Alici

**Affiliations:** Applied Mechatronics and Biomedical Engineering Research (AMBER) Group, University of Wollongong, Wollongong, NSW 2522, Australia; shenz@uow.edu.au (S.Z.); hzhou@uow.edu.au (H.Z.); rt910@uowmail.edu.au (R.T.)

**Keywords:** sEMG, pFMG, dataset, human–machine interface, wearable system, hand gesture recognition

## Abstract

Reliable hand gesture recognition (HGR) using wearable sensors remains challenging due to variability in arm posture, motion, and individual muscle activation patterns. To facilitate systematic investigation of multi-modal sensing strategies under realistic operating conditions, this paper presents a comprehensive dataset of surface electromyography (sEMG) and pressure-based force myography (pFMG) signals. The dataset includes three complementary subsets acquired under controlled static arm posture, multiple static arm postures, and combined static and dynamic arm postures. Signals were recorded using a custom-designed co-located sEMG-pFMG armband, enabling the simultaneous capture of electrical muscle activation and mechanical muscle deformation. System validation is conducted from three perspectives. First, hardware-level signal quality is assessed through signal-to-noise ratio (SNR) analysis across all gestures and sensing channels, demonstrating stable and reliable signal acquisition. Second, representative raw waveform examples are provided to qualitatively illustrate modality-specific and condition-dependent signal characteristics under static and dynamic arm-posture scenarios. Third, reproducible baseline gesture recognition experiments are performed using conventional machine learning classifiers. By providing multi-modal data acquired under both static and dynamic arm-posture conditions, along with clearly de-fined experimental protocols and baseline benchmarks, this dataset serves as a valuable resource for developing, evaluating, and comparing gesture recognition algorithms and arm-wearable human–machine interface (HMI) systems.

**Dataset:** https://www.kaggle.com/datasets/uowmultimodalhgr/semg-pfmg-multimodal-gesture-data

**Dataset License:** Apache 2.0

## 1. Introduction

Accurate and robust recognition of hand gestures from surface electromyography (sEMG) signals is crucial for the development of intuitive human–machine interfaces (HMIs), particularly in applications such as prosthetic limb control [1,2,3,4], powered exoskeletons [5,6,7,8,9,10,11], and assistive robotic systems [12,13,14]. sEMG records the electrical activity of muscles during contraction, representing the summation of motor unit action potentials (MUAPs) triggered by neural excitation of muscle fibers [15]. Typically, sEMG signals occupy the frequency range of 10–500 Hz, with the most informative physiological components concentrated between 20 and 150 Hz [16]. These signals serve as valuable indicators of muscle activation intensity [17,18,19], fatigue progression [20,21,22,23], neuromuscular coordination [24,25,26], and motion dynamics [27,28,29]. However, sEMG-based systems face persistent challenges due to the inherently low signal-to-noise ratio (SNR), especially during fine or low-force muscle activations [30,31,32]. Variations in upper-limb biomechanics, electrode placement, and external mechanical interactions further introduce signal inconsistencies [30,33,34]. Similar limitations are observed across other uni-modal sensing techniques used in HMIs, each being susceptible to modality-specific disturbances and sensitivity constraints [35].

To address these challenges, multi-modal sensing approaches have been increasingly explored, combining sEMG with complementary biosignals, including mechanomyography (MMG) [36,37,38], inertial measurement units (IMUs) [39,40,41,42], force myography (FMG) [43,44,45] and other systems to enhance robustness and reliability. Among these, FMG has gained particular attention because it measures the mechanical deformation of the forearm surface resulting from muscle contraction [46]. A pressure-based variant, known as pressure-based force myography (pFMG), utilizes compliant air chambers coupled with miniature pressure sensors distributed around the forearm to capture the volumetric fluctuations associated with muscular activation [43]. This technique offers a non-electrical and deformation-sensitive complement to sEMG, providing additional and complementary information. Integrating sEMG and pFMG into a co-located configuration, where both modalities are recorded from the same anatomical regions, provides an opportunity to observe electrical excitation and mechanical deformation simultaneously [43]. Our previous studies have demonstrated that the fusion of these signals enhances gesture recognition accuracy and robustness compared to using sEMG or FMG alone [34,43,47].

Despite the growing interest in hybrid sensing, publicly available multi-modal datasets remain limited, especially those capturing synchronized electrical and mechanical signals from the same anatomical locations. Existing open datasets such as Ninapro [48] and CapgMyo [2] have substantially advanced sEMG research, but they primarily provide single-modality EMG recordings collected under fixed or quasi-static arm postures [15,49]. As a result, most publicly shared EMG datasets are optimized for algorithm benchmarking rather than real-world deployment scenarios. In the context of wearable HMI development, several multi-modal datasets have been released, yet these are largely restricted to sEMG combined with inertial sensors such as accelerometers or IMUs. IMU+sEMG datasets [49,50] have been useful for capturing gross arm movements and orientation changes, and they are among the most common resources used for gesture recognition in wearable devices. However, IMUs measure motion rather than muscle deformation. As a result, these datasets fail to incorporate the mechanical aspect of muscle deformation, thereby restricting their ability to provide complementary information based on the integration of electrical and mechanical responses. Furthermore, even IMU-enhanced dataset [49] typically recorded only a small number of static arm configurations or short movement sequences, offering limited insight into how biosignals behave across continuous arm-position transitions. Table 1 summarizes the publicly available datasets for upper-limb gesture recognition and wearable HMI research.

Furthermore, another challenge in the field is the lack of standardized protocols for evaluating wearable HMI systems [48]. Gesture sets, sensor placements, data segmentation strategies, and evaluation metrics vary greatly across studies, making it difficult to reproduce results or compare algorithms fairly [32,58]. While datasets such as Ninapro [48] have become established benchmarks for sEMG only, there is no equivalent standard for hybrid or co-located multi-modal sensing systems. The absence of unified frameworks for experimental design and evaluation has slowed methodological progress and restricted the development of algorithms capable of generalizing across users, postures, and sensing configurations [32]. Creating open, multi-modal datasets with well-documented protocols is essential for enabling fair comparison, promoting reproducibility, and supporting systematic advancement in wearable HMI research.

Another persistent gap relates to the limb-posture effect, which causes the substantial changes in signal characteristics when the arm orientation varies [59,60]. Differences in muscle geometry, electrode alignment, tissue displacement, and skin tension across postures can produce large shifts in signal distributions [39,45,61]. Although the limb-posture effect has been widely reported, publicly available datasets addressing this challenge are typically limited to a few discrete static postures and often rely solely on EMG signals or with IMUs [33,39,62,63,64,65]. To our knowledge, none provides synchronized co-located sEMG-pFMG recordings under both static and dynamic arm-movement conditions.

To address these limitations, we present a collection of three open multi-modal datasets acquired using a custom-designed eight-pair co-located sEMG-pFMG armband [43]. Together, these datasets form a unified resource for studying static, multi-posture, and dynamic upper-limb gesture recognition under consistent hardware and protocol conditions:

(1) Dataset 1—Single Static Arm Posture: Seven gestures (relax, open, close, tripod, key, pronation, and supination) performed under a single fixed posture, recorded at 1000 Hz. This dataset provides a stable baseline for evaluating multi-modal signal characteristics and fusion strategies under minimal variability.
(2) Dataset 2—Three Static Arm Postures: Nine gestures (adding point and pinch) executed across three distinct static postures, including arm relaxing, 90° elbow flexing, and 90° arm extended forward, recorded at 2000 Hz. This dataset enables analysis of multi-modal behavior under various posture variations.
(3) Dataset 3—Static and Dynamic Arm Postures: Recordings contained three static postures and three dynamic transitions, captured at 2000 Hz. This dataset supports investigation into dynamic-static transfer learning, temporal modeling, and motion-robust multi-modal gesture recognition.

Across all three datasets, the sensor layout, acquisition system, gesture definitions, and labeling scheme were kept consistent to allow direct comparison. Each dataset provides synchronized sEMG and pFMG channels, detailed metadata, and standardized experimental documentation to facilitate reproducibility and cross-study benchmarking.

Collectively, this dataset series represents the first openly available resource to provide co-located sEMG-pFMG recordings across single-posture, multi-posture, and dynamic arm-movement conditions, addressing a critical gap in multi-modal wearable HMI research. These datasets offer a scalable foundation for studying sensor fusion, cross-position adaptation, movement-robust feature design, and domain-generalization strategies, contributing to the development of reliable and practical wearable upper-limb HMIs.

## 2. Dataset Description

All datasets are publicly available https://www.kaggle.com/datasets/uowmultimodalhgr/semg-pfmg-multimodal-gesture-data and are organized in a structured directory hierarchy that separates raw signal recordings, trial indices, condition labels, and metadata files.

**Figure 1:**
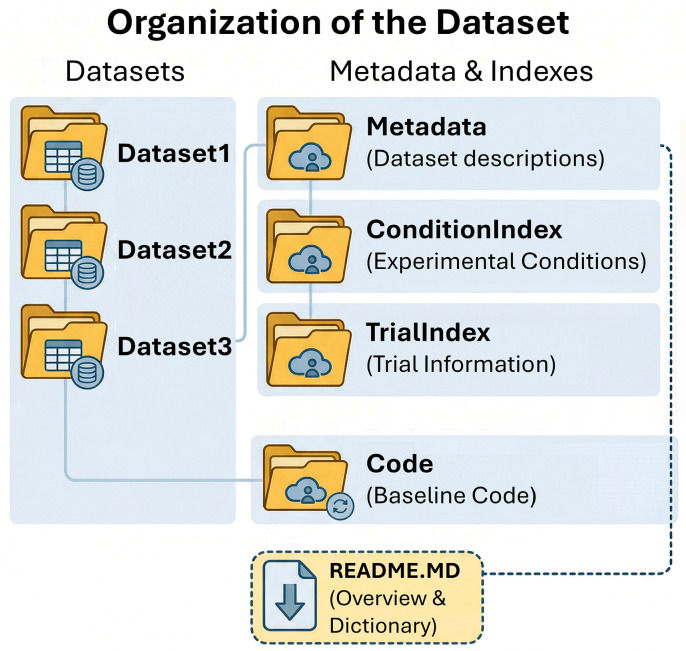
Overview of the dataset organization and file structure for the dataset. The database is organized into hierarchical directories, including Dataset1, Dataset2, and Dataset3, which store raw subject-wise recordings, along with Metadata, TrialIndex, and ConditionIndex folders that provide auxiliary information for data interpretation.

The complete dataset repository (Figure 1) contains three datasets (Dataset 1–3) collected using a co-located sEMG-pFMG armband system under progressively more complex arm-posture setups. Dataset 1 was acquired under a single controlled static arm posture, Dataset 2 includes three static arm postures spanning a representative daily range of upper-limb postures, and Dataset 3 extends these static conditions by incorporating dynamic transitions between arm postures. Raw signal recordings are stored in NumPy binary format (.npy), while trial boundary indices, condition labels, and auxiliary metadata are provided in separate index and metadata files to facilitate reproducible data loading and analysis.

### 2.1. Raw Data Files

For all three datasets, each raw data file corresponds to a single subject and is stored in the directories Dataset1/, Dataset2/, or Dataset3/, respectively. Each file has the following format:

(1) $$\left(n_{gestures},{\;n}_{channels},{\;n}_{samples}\right)$$

where $n_{gestures}$ denotes the number of hand gestures included in the dataset, $n_{channels}$ is the number of sensor channels, and $n_{samples}$ represents the total number of samples obtained by concatenating fixed-length trial windows along the time dimension.

The sensing system includes up to 16 channels arranged as eight co-located sEMG-pFMG pairs, where even-indexed channels correspond to sEMG signals and odd-indexed channels correspond to pFMG signals. For some subjects in Dataset 2, only seven co-located pairs (14 channels) are available due to hardware constraints; channel availability information for these subjects is explicitly documented in Metadata/dataset2_channel_availability.csv. The third dimension of each raw data array contains concatenated raw trial windows in their original acquisition order. No sliding-window segmentation or feature extraction has been applied to the raw recordings.

Dataset 1 contains recordings of seven hand gestures collected under a single static arm posture. Signals were sampled at 1000 Hz, and each trial has a duration of 2 s (2000 samples). Each gesture was repeated 8 or 9 times depending on the subject. Subject-specific repetition counts are documented in Metadata/dataset1_subject_reps.csv.

Dataset 2 contains recordings of nine hand gestures collected under three static arm postures (P1, P2, and P3). Signals were sampled at 2000 Hz, and each trial has a duration of 1 s (2000 samples). For each gesture, 12 trials were recorded, corresponding to four repetitions at each arm posture. All subjects share the same trial structure, while channel availability differs across subjects as documented in Metadata/dataset2_channel_availability.csv.

Dataset 3 contains recordings of nine hand gestures collected under both static and dynamic arm-posture conditions. Signals were sampled at 2000 Hz, and each trial has a duration of 1 s (2000 samples). For each gesture, 45 trials are provided, comprising 30 static-posture trials followed by 15 dynamic arm-posture transition trials. Trials are ordered in two consecutive blocks (static first, dynamic second), and trial-level condition labels are provided in the corresponding condition index file.

### 2.2. Trial Index Files

Trial boundaries for reconstructing individual trials from the concatenated raw recordings are provided in the Trial Index/directory. Each trial index file specifies the start and end sample indices for fixed-duration trials and has the format:

(2) $$\left(n_{gestures},n_{trials},2\right)$$

where the last dimension corresponds to the inclusive start and exclusive end indices of each trial.

For Dataset 1, subject-specific trial index files are provided due to variations in the number of trials across subjects. For Datasets 2 and 3, all subjects share identical trial structures; therefore, a single shared trial index file is provided for each dataset.

### 2.3. Condition Index Files

Trial-level experimental conditions are provided in comma-separated value (CSV) format in the ConditionIndex/directory. These files associate each trial with its corresponding arm posture or dynamic transition condition. For Dataset 2, the condition index specifies the static arm posture (P1, P2, or P3) associated with each trial. For Dataset 3, the condition index distinguishes between static arm-posture trials and dynamic arm-posture transition trials and provides labels for both static postures and dynamic transitions. Dynamic transition labels are further defined in Metadata/dynamic_transition_labels.csv, which specifies the start and end arm postures associated with each transition label.

### 2.4. Metadata Files

Additional metadata files are provided in the Metadata/directory to support data interpretation and reuse. These include global and dataset-specific gesture definitions, arm posture labels, dynamic transition definitions, channel mappings, participant information, and detailed acquisition parameters. Together, these metadata files enable transparent reconstruction of experimental conditions and facilitate reproducible analysis across datasets.

### 2.5. Code File

The code file for baseline HGR is provided, including script for data loading, data preprocessing, feature extraction, and baseline ML models (Random Forest (RF) and Linear Discriminant Analysis (LDA)). The provided code enables full reproduction of the results reported in this paper and serves as a reference implementation for future studies.

## 3. Methods

### 3.1. Participants

A total of 17 healthy adult volunteers participated across the three datasets included in this data collection. All participants were right-handed and reported no history of neuromuscular disorders, musculoskeletal injuries, or conditions affecting upper-limb motor function at the time of recording. All recordings were performed using the dominant upper limb (right hand), except for one participant (S09) whose data was collected from the left hand. This exception was intentionally included to facilitate investigations involving the non-dominant or less frequently used hand. Participant information recorded for this dataset includes age, sex, handedness, and the tested hand, in accordance with ethical approval and privacy considerations. All participant-related information is provided in anonymized form within the accompanying metadata file (participants.csv).

The three datasets (Table 2) were acquired using partially overlapping participant groups. Dataset 1, which focuses on gesture execution under a single static arm posture, includes recordings from 14 participants. Dataset 2, which introduces multiple static arm postures, includes recordings of 10 participants. Dataset 3, which introduces multiple static arm postures and dynamic arm-position transitions, includes recordings of 11 participants. A subset of participants contributed data to all three datasets, enabling direct comparison across experimental conditions. In addition, new participants were introduced in Datasets 2 and 3 to expand subject diversity under multi-posture and dynamic protocols. These three datasets can support cross-dataset and cross-condition evaluation, including studies that investigate model generalization across different recording contexts.

Data acquisition for the three datasets was conducted over a period spanning several months, rather than within a single recording session. This temporal separation introduces natural variability associated with long-term use, such as changes in sensor placement, skin conditions, and participant familiarity. As a result, the dataset may be useful for evaluating long-term stability, temporal robustness, and cross-session generalization of wearable HMI algorithms.

All participants provided written informed consent prior to participation. The study protocol was reviewed and approved by the University of Wollongong Human Research Ethics Committee (Approval No. 2023/006) following the rules of the Declaration of Helsinki of 1975. All data were fully anonymized prior to public release, and no personally identifiable information is included in the dataset.

### 3.2. Data Acquisition System

**Figure 2:**
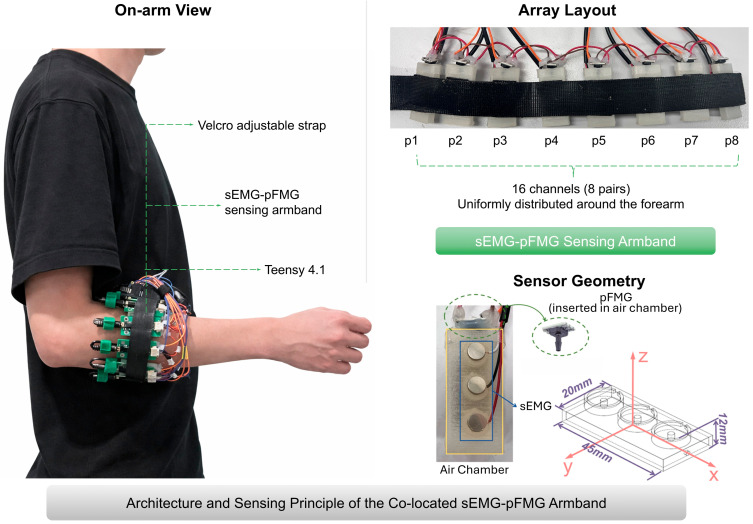
Architecture and sensing principle of the proposed wearable armband.

All datasets were acquired using a custom-built wearable armband integrating eight co-located sEMG-pFMG sensing pairs, resulting in a total of 16 synchronized signal channels (Figure 2). Each sensing pair was composed of a surface electromyography (sEMG) electrode and a pressure-based force myography (pFMG) sensor positioned in close spatial proximity, enabling simultaneous capture of electrical muscle activation and corresponding mechanical deformation from the same forearm region. Synchronization between sEMG and pFMG signals was achieved using a microcontroller-based data acquisition system (Teensy 4.1), where all channels were sampled under a shared system clock. The analog signals were acquired sequentially using a multiplexed analog-to-digital converter (ADC) across 16 channels, resulting in a consistent and minimal inter-channel sampling delay. To ensure reliable electrical conduction, circular stainless-steel electrodes with a radius of 3 mm and a thickness of 2 mm were used for sEMG acquisition. Each sensing unit incorporated an air chamber with overall dimensions of 45 mm × 20 mm × 12 mm, resulting in a compact form factor. The air chamber was 3D-printed by thermoplastic polyurethane (TPU), which was used to measure pressure variations associated with muscle contraction. This integrated design allows both the sEMG electrodes and the pFMG sensing elements to be housed within a single module, making the system compatible with the internal mounting constraints of commercially available prosthetic hands.

To capture pressure variations within each air chamber during muscle contraction, a miniature pressure transducer (ABPDANT015PGAA5, 0–15 psi gauge range, 0.25% accuracy; Honeywell International Inc., Pune, MA, USA) was embedded laterally into the chamber wall. This placement allowed the internal air pressure to respond directly to changes in forearm surface deformation associated with different hand gestures. The arm chamber was 3D-printed using flexible NinjaFlex TPU 85A (NinjaTek (Fenner Drives), Manheim, PA, USA), providing sufficient elasticity to maintain stable sensor-skin contact while accommodating differences in forearm size across participants. The eight sensing pairs were evenly distributed around the circumference of the mid-forearm, positioned approximately 6–10 cm distal to the elbow crease. The sensor modules were mounted onto a Velcro-based adjustable strap system, allowing individual sensing units to be repositioned as needed. Each air chamber was affixed to the strap using adhesive bonding, enabling rapid adjustment of sensor spacing and overall armband size while maintaining circumferential alignment.

Signal acquisition was performed using a Teensy 4.1 microcontroller board equipped with an ARM Cortex-M7 processor (ARM, Cambridge, UK). The sEMG signals were acquired using commercial OYMotion (Shanghai, China) analog EMG sensors with an amplification gain of 1000. For the pFMG modality, the Honeywell ABPDANT015PGAA5 pressure sensors incorporate an internal signal-conditioning circuit and provide an analog voltage output proportional to the measured pressure. No additional external analog amplification was applied prior to the analog-to-digital conversion. All 16 channels of sEMG and pFMG signals were sampled simultaneously through analog inputs to ensure temporal alignment between modalities. Digitized data were transmitted in real time to a host computer via a micro-USB serial connection for storage and monitoring. The low-profile design of the acquisition hardware facilitated integration with the wearable setup while minimizing motion restriction during both static and dynamic recordings.

### 3.3. Signal Processing

Following analog-to-digital conversion, the sEMG signals were digitally filtered to improve signal quality. Although sEMG activity spans a broad frequency range, the physiologically informative components are predominantly concentrated between 20 Hz and 150 Hz [16]. Accordingly, a second-order Butterworth high-pass filter with a cut-off frequency of 20 Hz was applied to suppress low-frequency motion artifacts, followed by a second-order Butterworth low-pass filter with a cut-off frequency of 150 Hz to remove high-frequency noise. In addition, a fourth-order digital notch filter (50 Hz) implemented as two cascaded second-order IIR sections, was applied to suppress mains power-line interference. This filtering strategy preserved the essential characteristics of the sEMG signals while minimizing common sources of noise encountered in wearable recordings. The pFMG signals exhibited a comparatively higher signal-to-noise ratio (SNR). As a result, no additional filtering was applied to the pressure channels, and the raw pressure signals were recorded directly through the data acquisition system.

Synchronization between sEMG and pFMG signals was inherently maintained during acquisition through a shared microcontroller-based data acquisition system (Teensy 4.1), where all channels were sampled under a common system clock. Regarding data annotation, labels were defined directly by the experimental protocol and the structured recording procedure. Each trial corresponded to a single gesture performed within a fixed recording window, and data acquisition was initiated only after the target gesture had been fully established. Consequently, each recorded segment inherently represents a steady-state gesture with a known label. During data storage (NumPy: .npy format), the signals were organized into a structured array with dimensions $\left(n_{gestures},\;\;n_{channels},\;\;n_{samples}\right)$, where each entry directly corresponds to a specific gesture class. Therefore, no post hoc relabeling or segmentation was required, and the temporal correspondence between signals and labels is inherently preserved.

All acquisition parameters, channel ordering, and sensor pairings are documented in the accompanying metadata files to ensure transparent interpretation and reuse of the dataset.

### 3.4. Acquisition Protocol

All recordings were conducted in an indoor environment using standardized acquisition procedures. Prior to formal data collection, participants underwent a brief familiarization session, during which they were guided through the required hand gestures and arm positions. This pre-training phase was designed to ensure that participants clearly understood the experimental instructions and could perform the gestures and postural configurations smoothly and accurately during recording. During the experiments, audio-based verbal cues were provided to indicate when participants should adopt specific arm postures and execute particular hand gestures. These cues ensured consistent timing and sequencing across participant. Participants were instructed to maintain a moderate and consistent level of muscle activation during gesture execution. Because a fixed recording window was used for each trial, data acquisition was initiated only after the target gesture had been fully presented, ensuring that no transition-related transient signals were included in the recorded data. As a result, each recorded segment corresponded to a stable gesture state with a well-defined label.

Ground truth labels were directly determined by the predefined experimental protocol and the corresponding recording structure. Each trial was recorded as an independent segment associated with a single gesture, and the dataset was organized in the form of $\left(n_{gestures},{\;n}_{channels},\;\;n_{samples}\right)$, where each segment inherently corresponds to a known gesture class. Therefore, no post hoc relabeling or segmentation was required. To ensure annotation reliability, all recording sessions were supervised by the experimenter, who verified correct gesture execution and adherence to the instructed arm positions.

Data acquisition and monitoring were implemented using Python 3.10.4 within Visual Studio Code. The experimental workstation was equipped with a 12th-generation Intel^®^ Core™ i7-12700H CPU (Santa Clara, CA, USA) and an NVIDIA GeForce RTX 3060 GPU (Santa Clara, CA, USA). Scheduled rest periods were incorporated throughout the protocols to reduce muscle fatigue and maintain consistent signal quality. No explicit sensor-level calibration was performed prior to data collection. For sEMG signals, the raw measurements inherently fluctuate around zero with both positive and negative components. To preserve the full temporal and amplitude information of the muscle activation patterns, no additional preprocessing or calibration (e.g., rectification or envelope extraction) was applied at the data acquisition stage. For pFMG signals, no calibration was applied either, as the study focuses on relative signal patterns and gesture-dependent variations. Feature extraction was performed during downstream analysis for both sEMG and pFMG, which are able to capture discriminative information for gesture recognition tasks. It is noted that due to variations in armband tightness and individual anatomical differences, the baseline signal level (under relax gesture) of pFMG may vary across subjects. While this does not affect intra-subject analysis, it may introduce variability in inter-subject scenarios. Therefore, for applications involving cross-subject generalization, it is recommended to perform a simple baseline calibration (e.g., offset normalization using the relaxed gesture under a standardized arm position) to improve consistency across participants.

#### 3.4.1. Dataset 1: Single Static Arm Posture

**Figure 3:**
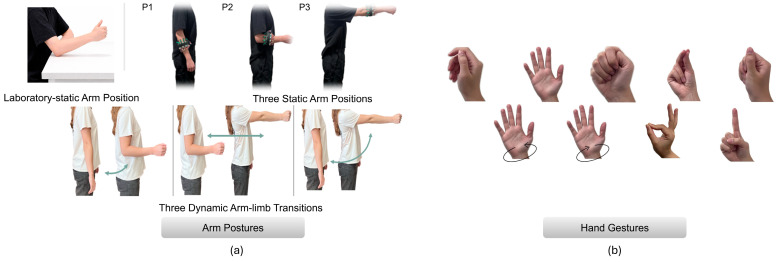
Arm postures and hand gestures. (**a**) Arm postures adopted during data acquisition. (**b**) Hand gesture set used for gesture recognition experiments. Nine representative hand gestures were selected to reflect a range of functional hand postures commonly used in daily activities, including relax, open, fist, tripod, key, pronation, supination, point, and pinch.

Dataset 1 was designed to establish a baseline dataset under a controlled and fixed arm posture (Figure 3a). Participants were instructed to perform seven hand gestures, including relax, open, fist, tripod, key, pronation, and supination, presented sequentially during each trial (Figure 3b). Each gesture was maintained for 2 s (2000 data points), followed by a 2 s rest period before the next gesture. Verbal cues were used to prompt participants when to transition between gestures. Each participant completed nine full repetitions of the entire gesture sequence. This dataset provides a controlled reference for analyzing multi-modal signal characteristics in the absence of arm-position variability.

#### 3.4.2. Dataset 2: Multiple Static Arm Postures

Dataset 2 extends the experimental design by introducing multiple static arm postures. Data were collected at a sampling rate of 2000 Hz using the same acquisition hardware. Participants were first familiarized with three predefined arm postures (Figure 3a):

(a) P1: arm relaxed alongside the body
(b) P2: arm positioned forward with the elbow flexed at approximately 90°
(c) P3: arm extended forward at shoulder height

A total of nine commonly used hand gestures were recorded: relax, open, fist, tripod, key, pronation, supination, pinch, and point (Figure 3b). The experiment was organized into two identical recording rounds, separated by a 5 min rest interval. This interval served multiple purposes: it reduced the accumulation of muscle fatigue, helped maintain signal stationarity within each round, and preserved overall data quality. At the same time, the temporal gap introduced a controlled separation between rounds, allowing assessment of whether the sensing system and signal characteristics remained stable over time. This design supports investigations into short-term temporal robustness, which is relevant for wearable HMI applications where sensors are expected to perform consistently across repeated use.

Each gesture trial consisted of 2000 samples, corresponding to a 1 s gesture hold, followed by a 3 s rest period. In each round, participants performed every hand gesture within each predefined arm posture, with two repetitions per gesture per posture, resulting in a total of six repetitions per gesture across all arm postures. After completing both rounds, the recordings were combined to form a single dataset, yielding a total of 12 repetitions per gesture across the three static arm postures.

Rather than repeatedly performing all repetitions of a gesture within a single fixed arm posture, the protocol was organized in a cycle-based manner. In each cycle, participants first adopted one arm posture and executed the full gesture set sequentially, presenting each gesture once. They then transitioned to the next arm posture and repeated the same gesture sequence, continuing until all three arm postures had been completed. Subsequent cycles restarted from the first arm posture and followed the same procedure. This design introduces subtle, natural variations in arm posture between cycles, even when the nominal arm posture is the same, reflecting the fact that users cannot precisely reproduce identical arm configurations in repeated trials.

This structure enables a wide range of downstream applications, including posture-invariant gesture recognition, evaluation under unknown or unseen arm postures, and robustness analysis across repeated use.

#### 3.4.3. Dataset 3: Static and Dynamic Arm Postures

Dataset 3 was developed to capture multi-modal signals under both static and dynamically changing arm postures, thereby reflecting more realistic usage conditions. All data were recorded at a sampling frequency of 2000 Hz. The same nine hand gestures as in Dataset 2 were used (Figure 3b).

For the static component, the same three arm postures (P1–P3) defined in Dataset 2 were adopted (Figure 3a). As in Dataset 2, data collection followed a cycle-based protocol, where one cycle consisted of executing the complete gesture set sequentially under a single arm posture before moving to the next arm posture. This process was repeated across cycles, allowing natural postural variation to occur between cycles even for nominally identical arm postures. Compared to Dataset 2, a larger number of repetitions were collected under static conditions, increasing the overall dataset size and providing richer coverage of inter-cycle variability. Static recordings were organized into two rounds, separated by a 5 min rest period, following the same rationale as Dataset 2. This design helps preserve signal quality by reducing fatigue while introducing controlled temporal separation, enabling analysis of signal stability, repeatability, and short-term temporal drift. In each round, each gesture was repeated five times per arm posture, yielding 15 repetitions per gesture across three static postures.

In addition to static recordings, Dataset 3 includes a dynamic component designed to capture continuous arm movement during gesture execution. Participants maintained each gesture while performing three predefined dynamic arm-movement postures, covering a representative range of elbow and shoulder motion encountered in daily activities (Figure 3a), including: P1 to P2, P2 to P3, and P1 to P3. These included transitions spanning different elbow and shoulder configurations to cover a representative range of daily arm movements. Participants were not constrained to a fixed movement speed. Instead, they were instructed to perform continuous and smooth motions that completed the full range of each movement pattern. Each dynamic sequence included at least one complete movement cycle, with additional cycles performed if time permitted within the recording window. The dynamic component was recorded in a separate round, with a rest period (5 min) provided after data collection under static arm postures to minimize fatigue. In the dynamic round, each gesture was repeated five times per arm posture, yielding 15 repetitions per gesture across three static postures.

Each gesture (under both static and dynamic arm postures) was recorded using a fixed window of 2000 samples, corresponding to a 1 s gesture presentation, followed by a 2 s rest period. Researchers monitored motion execution to ensure smooth transitions and full completion of the intended movement patterns. After completing all recording rounds, including both static and dynamic conditions, the data were combined to form a single unified dataset. For each gesture, the dataset contains 45 repetitions in total, comprising the first 30 repetitions recorded under the three static arm postures and the subsequent 15 repetitions recorded under the three dynamic arm-posture conditions.

By combining increased repetition counts, cycle-based static recordings, and continuous dynamic arm movements, Dataset 3 provides a substantially larger and more diverse dataset. This design supports advanced investigations into motion-robust gesture recognition, adaptation to unknown arm postures, dynamic-static transfer learning, and the evaluation of multi-modal sensing systems under realistic and time-varying conditions.

## 4. Technical Validation

This section provides a comprehensive technical validation of the released datasets from three complementary perspectives: hardware-level signal quality, qualitative signal characteristics under varying arm-posture conditions, and baseline gesture recognition performance. First, the signal-to-noise ratio (SNR) is analyzed to assess the stability and reliability of the co-located sEMG-pFMG sensing hardware. Second, representative raw waveform examples are presented to qualitatively illustrate the modality-specific and condition-dependent signal behaviors under static and dynamic arm postures. Finally, reproducible baseline gesture recognition experiments are conducted using standard machine learning (ML) pipelines to demonstrate the practical usability of the datasets for downstream analysis. Together, these evaluations aim to verify that the datasets provide reliable signal quality, meaningful physiological and mechanical information, and well-defined experimental protocols suitable for developing and benchmarking multi-modal HGR methods.

### 4.1. Hardware-Level Signal Quality Assessment

To evaluate the hardware-level signal quality of the proposed co-located sEMG-pFMG armband, the SNR was calculated for all sensing channels across all gestures in Dataset 1 using a representative subject. This analysis focuses on characterizing the distribution and variability of SNR values across different gestures/channels. To quantitatively evaluate signal quality, the SNR was calculated for each sensing channel and each gesture using [66]:

(3) $${SNR}_{dB}=20\log_{10}\left(\frac{A_{signal}}{A_{noise}}\right)$$

where $A_{signal}$ and $A_{noise}$ denote the root mean square (RMS) amplitudes of the signal and noise segments, respectively. The noise segment was obtained from the full 2000-sample (one repetition) resting trial immediately preceding each gesture, while the signal segment corresponded to the full 2000-sample (one repetition) recording of the performed gesture. The SNR was calculated independently for each sensing channel and each gesture.

**Figure 4:**
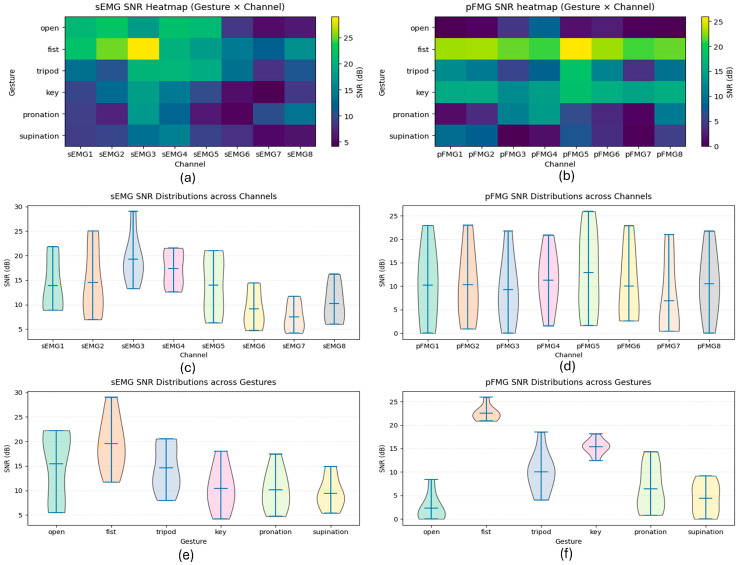
SNR characterization of the co-located sEMG-pFMG dataset. (**a**) sEMG SNR heatmap showing gesture-channel-dependent variations in signal quality across all sEMG sensing channels. (**b**) pFMG SNR heatmap showing gesture-channel-dependent variations in signal quality across all pFMG sensing channels. (**c**) Channel-wise violin plots of sEMG SNR values aggregated across all gestures, highlighting channel-dependent SNR distributions. (**d**) Channel-wise violin plots of pFMG SNR values aggregated across all gestures, reflecting spatial variability in mechanical signal sensitivity. (**e**) Gesture-wise violin plots of sEMG SNR values across all channels, demonstrating gesture-dependent differences in electrical muscle activation. (**f**) Gesture-wise violin plots of pFMG SNR values across all channels, capturing variations in pressure-based mechanical responses across gestures.

Figure 4a,b present the SNR heatmaps for sEMG and pFMG signals, respectively, where each row corresponds to a gesture and each column represents an individual sensing channel. Clear variations in SNR can be observed across both gestures and channels for both modalities. It is worth noting that low SNR values are observed for certain gesture-channel combinations in both modalities. This behavior is expected in multi-channel sEMG-pFMG acquisition, as not all muscles or pressure regions are actively engaged during every gesture. Such variability reflects the inherently gesture-dependent nature of muscle activation and the channel-location-dependent sensing characteristics of the armband. In particular, certain channels exhibit higher SNR for gestures that strongly engage the underlying muscle groups or induce larger mechanical deformation, while lower SNR values appear in gesture-channel combinations associated with weaker activation or minimal pressure variation.

To further illustrate the distribution of SNR values, violin plots are shown for both modalities. Figure 4c,d depict the SNR distributions across channels for each gesture, highlighting that different gestures give rise to distinct SNR distributions due to differences in activation patterns and mechanical interaction with the armband. In addition, Figure 4e,f present channel-wise violin plots, summarizing the SNR distributions of each sensing channel across all gestures. These plots demonstrate that all channels produce measurable signals across multiple gestures, while exhibiting different SNR ranges and distribution shapes, indicative of channel-specific sensitivity and spatial heterogeneity. From the violin plots, the mean SNR values across both gestures and channels are well above the minimum acceptable SNR level (1.2 dB) [67], indicating that the acquired signals provide a sufficient SNR margin at the hardware level and supporting the reliability of the data acquisition system.

### 4.2. Representative Waveform Examples Under Static and Dynamic Arm-Posture Conditions

**Figure 5:**
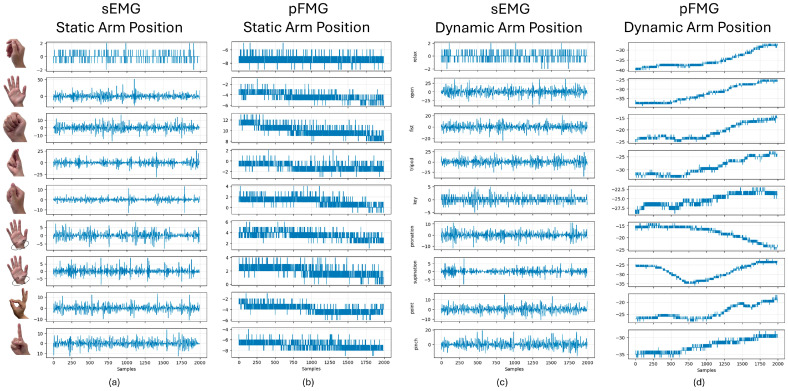
Representative sEMG and pFMG waveform examples under static and dynamic arm-posture conditions. (**a**) sEMG recordings acquired at a static arm posture. (**b**) pFMG recordings acquired at a static arm posture. (**c**) sEMG recordings acquired during a dynamic arm-posture condition. (**d**) pFMG recordings acquired during a dynamic arm-posture condition. For each condition, one representative trial is included.

To qualitatively illustrate the effects of arm-posture conditions on the acquired signals, representative raw waveform examples from Dataset 3 are presented for both static and dynamic arm-posture scenarios. Figure 5 shows sEMG and pFMG signals recorded from a single representative subject using one co-located sEMG-pFMG channel pair, with one trial selected for a static arm posture and one trial selected for a dynamic arm-posture transition. All gestures are included to demonstrate the consistency of condition-dependent signal characteristics across different hand configurations.

Under static arm-posture conditions, the sEMG signals (Figure 5a) exhibit gesture-dependent temporal patterns and amplitude variations. Different hand gestures produce distinct sEMG waveform characteristics, reflecting variations in muscle recruitment strategies and activation intensity across gestures. In contrast, the corresponding pFMG signals under static conditions (Figure 5b) are comparatively more stable in their temporal profiles. Nevertheless, the absolute pFMG signal levels still vary across gestures, which can be attributed to differences in muscle bulging and tissue deformation induced by gesture-specific force generation. These observations highlight the complementary sensing characteristics of the two modalities: sEMG primarily captures electrical muscle activation driven by neural excitation, while pFMG reflects mechanical deformation associated with muscle contraction and force transmission.

When the arm posture becomes dynamic, gesture-dependent differences in sEMG signal (Figure 5c) patterns remain observable. However, from visual inspection, the overall sEMG signal characteristics do not exhibit substantial changes compared to the static arm-posture condition. This suggests that sEMG is relatively insensitive to arm postures and continues to predominantly encode muscle activation rather than global limb movement. In contrast, the pFMG signals (Figure 5d) show markedly different behavior under dynamic arm-posture conditions. Pronounced temporal trends and gradual baseline shifts are observed across gestures, with pFMG signals varying continuously in correspondence with arm movement. This behavior arises from changes in muscle geometry, soft tissue redistribution, and contact pressure between the forearm and the armband during dynamic motion. As the arm posture evolves, the relative positioning between the sensing elements and underlying tissues changes, leading to systematic variations in the measured pressure signals. These representative waveform examples clearly demonstrate the higher sensitivity of pFMG to arm-posture dynamics, while reinforcing the modality-specific roles of sEMG and pFMG in capturing electrical and mechanical aspects of muscle activity.

Overall, these representative waveform examples highlight the complementary nature of sEMG and pFMG signals. While sEMG predominantly captures gesture-dependent electrical muscle activation with limited sensitivity to arm-posture changes, pFMG provides mechanically informative signals that are highly responsive to arm posture and movement. This complementarity motivates the inclusion of both static and dynamic arm-posture conditions in the released dataset. By encompassing a wide range of postural and motion scenarios, the dataset enables the investigation of gesture recognition and muscle activity modeling methods under realistic conditions, where arm posture variability is unavoidable in practical HMI applications.

### 4.3. Baseline Hand Gesture Recognition

For all datasets, a consistent feature extraction and classification pipeline was adopted to provide reproducible baseline benchmarks. Raw signals were segmented using a sliding window of 200 ms with a stride of 10 ms. For sEMG signals, a standard set of time-domain features was extracted, including root mean square (RMS), variance (VAR), simple square integral (SSI), mean absolute value (MAV), average amplitude change (AAC), and difference absolute standard deviation value (DASDV). For pFMG signals, only the RMS feature was used. The data selection was following our previous study [43]. Feature vectors from sEMG and pFMG were evaluated separately as well as in a fused representation formed by concatenation. Two widely used conventional classifiers, RF and LDA, were employed as baseline models. All experiments followed fixed train–test splits specific to each dataset to preserve the temporal structure of the trials and ensure reproducibility. This split strategy was adopted to avoid potential data leakage that may arise from random percentage-based splitting, where highly correlated samples from the same trial or repetition could be simultaneously included in both training and testing sets.

**Figure 6:**
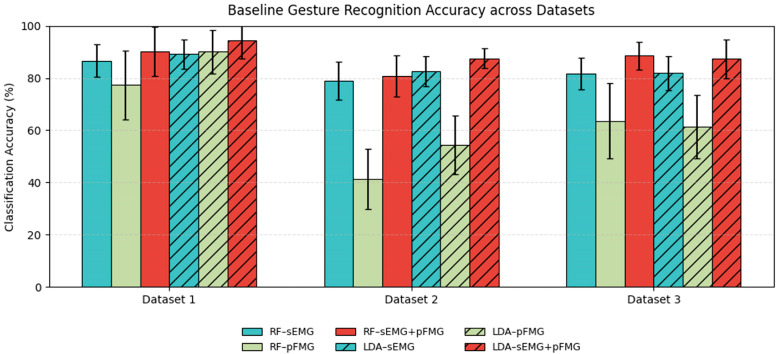
Baseline hand gesture recognition accuracy across datasets.

Baseline gesture recognition results obtained using RF and LDA are summarized in Figure 6. All results are reported as mean classification accuracy across subjects. For Dataset 1, subjects performed 8–9 trials of each gesture. To maintain consistency across subjects, the first five trials were used for training, and the subsequent three trials were used for testing for all participants. Under this controlled static setting, fusing sEMG and pFMG features consistently improves performance for both classifiers, yielding the highest mean accuracies. For Dataset 2, a fixed trial-based split was employed, with the first eight trials used for training and the remaining four trials used for testing. Compared to Dataset 1, increased variability in acquisition conditions leads to a noticeable reduction in pFMG-only performance, particularly for RF, while sEMG-based recognition remains comparatively stable. However, multi-modal fusion of sEMG and pFMG features consistently achieves higher mean classification accuracies than the single-modality baselines for both classifiers, highlighting the benefit of combining electrical and mechanical sensing under more challenging conditions. For Dataset 3, which explicitly incorporates both static and dynamic arm-posture data, the training set consisted of the first 15 static trials together with 15 dynamic arm-posture trials, while the remaining 15 static trials were used for testing. This split design evaluates the ability of learned models to generalize across arm-posture variability. The fusion of sEMG and pFMG features results in higher and more stable recognition accuracies compared to either modality alone for both RF and LDA.

To statistically evaluate the effectiveness of multi-modal fusion, pairwise comparisons between the multi-modal fusion approach and each individual modality were performed separately for each dataset and classifier using two-sided paired Student *t*-tests (Table 3). Statistical significance was defined as *p* < 0.05. Significant improvements over the single-modality approaches were observed in 10 of the 12 comparisons (*p* < 0.05). For the remaining two comparisons (Dataset 1 using LDA and Dataset 2 using RF), multi-modal fusion still achieved higher mean classification accuracies than the sEMG-only approach, although the improvements did not reach statistical significance (*p* = 0.11 and *p* = 0.40, respectively).

Overall, the baseline results across all three datasets confirm that the released datasets support reproducible gesture recognition experiments using standard ML pipelines. More importantly, multi-modal sEMG-pFMG fusion consistently achieved higher mean classification accuracies than the individual modalities across different datasets, classifiers, and train–test protocols. Pairwise statistical comparisons further confirmed that these improvements were statistically significant in the majority of comparisons, reinforcing the complementary nature of electrical and mechanical sensing modalities. By providing data collected under both static and dynamic arm-posture conditions with clearly defined trial-based splits, the dataset enables systematic investigation of multi-modal gesture recognition methods under realistic and variable operating conditions.

The baseline conventional ML models presented in this work are intended to provide simple and reproducible reference benchmarks for validating the released dataset. The accompanying baseline implementation provides a complete reference workflow, including data loading, preprocessing, feature extraction, train–test splitting, and evaluation, thereby facilitating the use of the released dataset by future researchers. The primary objective of this study is to introduce and technically validate a publicly available multimodal dataset for the research community. To further demonstrate that the proposed co-located sEMG-pFMG wearable platform and data collection protocol are suitable for advanced DL research, representative results from our previous studies are summarized in Table 4. These studies employed DL approaches using the same sensing platform and experimental protocols, demonstrating the capability of the proposed framework to support the development and evaluation of advanced DL algorithms.

## 5. User Notes

The released datasets are intended for research and educational purposes related to HGR, HMIs, and multi-modal biosignal analysis. Users may employ the datasets to develop, evaluate, and benchmark signal processing and ML methods under static and dynamic arm-posture conditions.

To ensure fair comparison and reproducibility, users are encouraged to follow the provided trial-based train–test split protocols and baseline evaluation procedures described in this paper. In particular, random percentage-based data splitting is discouraged, as temporally adjacent samples within the same trial are highly correlated and may lead to data leakage.

The dataset supports independent use of sEMG or pFMG signals as well as multi-modal fusion. Researchers may further extend the dataset for cross-subject evaluation, robustness analysis under arm-position variability, or integration with advanced ML models.

For users wishing to perform baseline offset normalization of the pFMG signals, a reference Python script (0_pFMG_Calibration.py) is provided in the Code folder of the released dataset repository. The script calculates the mean pFMG value of the corresponding relax repetition for each sensing channel and subtracts this channel-specific baseline from the corresponding repetition of all gesture recordings. This reference implementation is provided to facilitate the use of the released dataset. Depending on the specific application, users may also adopt alternative preprocessing or normalization strategies appropriate for their own research objectives.

The three datasets were designed and collected independently to provide progressively more challenging recording conditions, from a single static arm posture to multiple static arm postures and dynamic arm movements. Researchers may therefore select the dataset that best matches their own research objectives. For applications involving multiple datasets, such as transfer learning, domain adaptation, or cross-dataset benchmarking, users are encouraged to first resample the recordings to a common sampling frequency appropriate for their specific application.

Any use of the dataset in scientific publications should cite this paper accordingly.

## 6. Discussion

Compared with existing publicly available sEMG datasets [2,48,49,50,51,52,53,54,55,56,57], the proposed dataset provides several distinctive features. While most existing datasets focus solely on sEMG signals collected under fixed arm postures, the proposed dataset synchronously records co-located sEMG and pFMG signals, enabling the investigation of multi-modal sensing strategies for wearable HMIs. Furthermore, by providing three complementary datasets covering single static, multiple static, and dynamic arm-posture conditions, the released dataset enables a systematic investigation of gesture recognition under progressively more realistic operating conditions. Together with the accompanying metadata, standardized acquisition protocol, and baseline validation results, the dataset provides a reproducible benchmark for the development, evaluation, and comparison of future multi-modal gesture recognition algorithms.

The technical validation results demonstrate that the sensing system provides stable and reliable signal acquisition across different gestures and recording conditions, while the representative waveform analysis highlights the complementary characteristics of electrical muscle activation and mechanical muscle deformation signals. In addition, the baseline gesture recognition experiments confirm that multi-modal fusion of sEMG and pFMG consistently improves recognition performance compared to single-modality approaches, particularly under more challenging posture-varying conditions. These findings support the potential of co-located multi-modal sensing for improving the robustness of wearable HMI systems in practical applications. Beyond gesture recognition, the dataset is also expected to facilitate future studies on posture-invariant learning, multi-modal fusion strategies, cross-subject generalization, adaptive wearable interfaces, dynamic movement analysis, and other advanced ML algorithms. This dataset provides a scalable foundation for future extensions involving long-term recordings, clinical populations, additional sensing modalities, and more complex daily movement scenarios.

## 7. Conclusions and Future Work

This paper presented the first publicly available co-located sEMG-pFMG dataset for hand gesture recognition under varying arm-position conditions. The released dataset comprises three complementary subsets covering single static, multiple static, and dynamic arm-posture scenarios, together with detailed metadata, standardized acquisition protocols, and baseline validation results. By providing synchronized electrical and mechanical muscle activity recordings under realistic operating conditions, the dataset is expected to serve as a valuable resource for a broad range of applications, including gesture recognition, multimodal wearable sensing, human–machine interaction, prosthetic control, rehabilitation, muscle activity modeling, and assistive technologies. In addition, the released dataset provides a standardized benchmark for the development, evaluation, and comparison of future multi-modal learning algorithms.

One limitation of the current dataset is that anthropometric measurements, such as forearm circumference, BMI, and muscle mass, were not collected. Future versions of the dataset will consider incorporating additional anthropometric measurements to further enhance the representativeness and research value of the released dataset. As part of our ongoing research efforts, we plan to continuously expand the proposed dataset to further enhance its value for the research community. Future work will focus on increasing the number and diversity of participants, incorporating additional hand gestures and task-specific activities, and extending data collection to clinical populations. We also intend to continuously improve the dataset by enriching its scale and diversity, providing a comprehensive public benchmark for multi-modal wearable sensing, human–machine interaction, and assistive technology research.

## 8. Specifications Table


**Subject**
Biomedical Engineering.
**Specific subject area**
Co-located sEMG-pFMG dataset for hand gesture recognition under Varying Arm-Position conditions.
**Type of data**
Raw data: 777 MB (.npy)
**Data collection**
Data were collected using a custom-built wearable armband comprising eight co-located sEMG-pFMG sensing pairs (16 channels). sEMG was acquired using stainless-steel electrodes and pFMG using Honeywell ABPDANT015PGAA5 pressure sensors, synchronized via a Teensy 4.1 microcontroller. Data acquisition was implemented in Python 3.10.4 (Visual Studio Code). Participants performed predefined hand gestures under single static, multiple static, and dynamic arm-posture conditions. sEMG signals were filtered (20–150 Hz, 50 Hz notch), whereas raw pFMG signals were preserved.
**Data source location**
The University of Wollongong, Wollongong, NSW 2522, Australia
**Data accessibility**
Repository name: sEMG+pFMG multimodal gesture data Direct URL to data: https://www.kaggle.com/datasets/uowmultimodalhgr/semg-pfmg-multimodal-gesture-data
**Related research article**
None.
**Previously published dataset or data descriptor**
None.

## Figures and Tables

**Table 1:** Publicly available datasets for upper-limb gesture recognition and wearable HMI research. Public datasets primarily use sEMG or sEMG+IMU; none co-located sEMG and mechanical sensing (FMG/pFMG) across static and dynamic postures.

Datasets	Modalities	Subjects	Gestures	Limb Postures	Repetitions
CapgMyo [2]	HD-sEMG	18	8	1 (static)	10
Ninapro [48]	sEMG, ACC	Dataset 1:27 Dataset 2: 40 Dataset 3: 11	Dataset 1: 53 Dataset 2: 50 Dataset 3: 50	1 (static)	Dataset 1: 10 Dataset 2: 6 Dataset 3: 6
Kyranou et al. [49]	sEMG	8	6	9 ^a^ (static)	10
Physical Action [50]	sEMG, IMU	1	N/A ^b^	4 ^c^ (3 static + 1 dynamic)	4
GRABMyo [51]	sEMG	43	16	1 (static)	7
ISRMyo-I [52]	sEMG	6	13	1 (static)	2 repetitions/day; 10 days
MeganePro [53]	sEMG, ACC	45	10	2 ^d^ (static)	8
putEMG [54]	sEMG	44	8	1 (static)	20 ^e^ (sequential repetitive)
MyoBit [55]	sEMG	24	8	2 (static)	3
SeNic [56]	sEMG	36 ^f^	7	3 (static)	3
GrabMyo [53]	sEMG	43	16	1 (static)	1 session/day; 3 days
CEMHSEY [57]	HD-sEMG	Part 1: 13 Part 2: 6	7 grasps; 11 gestures	1 (static)	Part 1: 2 repetitions/day; 11 days Part 2: 5 repetitions/day; 11 days
This Work	sEMG, pFMG	Dataset 1: 14 Dataset 2: 10 Dataset 3: 11	Dataset 1: 7 Dataset 2: 9 Dataset 3: 9	Dataset 1: 1 (static) Dataset 2: 3 (static) Dataset 3: 6 (3 static + 3 dynamic)	Dataset 1: 9 Dataset 2: 4 repetitions/limb position Dataset 3: 15 repetitions/limb position (10 static + 5 dynamic)

^a^ Five distinct arm postures were recorded on each day, with only one same arm posture shared between the two recording days. ^b^ No gesture-specific data were recorded; the dataset consists solely of routine daily activities. ^c^ Four routine daily activities were recorded, comprising three static postures and one dynamic activity. ^d^ This refers to recordings acquired under both seated and standing postures. ^e^ The dataset includes seven long repetitions, seven short repetitions, and six sequential gesture repetitions, resulting in a total of 20 repetitions per gesture. ^f^ The dataset was assembled from recordings obtained across multiple participant subsets, each contributing to distinct components of the data collection protocol.

**Table 2:** Dataset summary.

	Dataset 1	Dataset 2	Dataset 3
Intact Subjects	14	10	11
Data Acquisition System	8 pairs (sEMG-pFMG)	8 pairs (sEMG-pFMG)	8 pairs (sEMG-pFMG)
Total Number of Movements (relax included)	7	9	9
Number of Repetitions	9	12	30 (static) 15 (dynamic)

**Table 3:** Pairwise statistical comparisons between multi-modal fusion and single-modality approaches using paired Student’s *t*-tests.

Dataset	Classifier	sEMG-pFMG vs. sEMG (*p*)	sEMG-pFMG vs. pFMG (*p*)
Dataset 1	RF	0.01	4.87 × 10^−5^
	LDA	0.11	1.79 × 10^−3^
Dataset 2	RF	0.40	6.28 × 10^−7^
	LDA	6.60 × 10^−3^	1.56 × 10^−6^
Dataset 3	RF	3.50 × 10^−5^	7.10 × 10^−5^
	LDA	0.02	5.65 × 10^−7^

**Table 4:** Representative DL studies using the proposed sensing platform and experimental protocol.

Study	Experimental Protocol	DL Model	Accuracy
[47]	3 static arm postures	Self-adaptive CNN	88.34%
[68]	3 static arm postures	GCN	84.69%
[69]	3 static + 3 dynamic arm postures	DFF-TCN	96.50%

## Data Availability

Dataset presented in this work is openly available at https://www.kaggle.com/datasets/uowmultimodalhgr/semg-pfmg-multimodal-gesture-data.

## Abbreviations

The following abbreviations are used in this manuscript:

AAC	Average amplitude change
ADC	Analog-to-digital converter
DASDV	Difference absolute standard deviation value
FMG	Force myography
HGR	Hand gesture recognition
HMI	Human–Machine Interface
IMU	Inertial measurement unit
LDA	Linear Discriminant Analysis
MAV	Mean absolute value
ML	Machine learning
MMG	Mechanomyography
MUAP	motor unit action potential
pFMG	Pressure-based force myography
RF	Random Forest
RMS	Root mean square
sEMG	Surface electromyography
SNR	Signal-to-noise ratio
SSI	Simple square integral
TPU	Thermoplastic polyurethane
VAR	Variance
